# HDG11 upregulates cell-wall-loosening protein genes to promote root elongation in *Arabidopsis*

**DOI:** 10.1093/jxb/eru202

**Published:** 2014-05-12

**Authors:** Ping Xu, Xiao-Teng Cai, Yao Wang, Lu Xing, Qiong Chen, Cheng-Bin Xiang

**Affiliations:** School of Life Sciences, University of Science and Technology of China, Hefei, Anhui 230027, China

**Keywords:** Cell-wall-loosening protein genes, cellulase, *edt1*, expansin, *HDG11*, pectin-related enzymes, XTH.

## Abstract

The gain-of-function mutant *edt1* shows significantly enhanced drought tolerance and a well-developed root system including deeper primary roots and more lateral roots. To explore the molecular mechanisms underlying the improved root system of *edt1*, we performed transcriptome comparison between the wild-type and *edt1* roots. One of the interesting findings from the analysis was that several gene families of cell-wall-loosening proteins were upregulated in the mutant roots, including expansins, extensins, xyloglucan endotransglucosylase/hydrolases (XTHs), pectin-related enzymes, and cellulases. Most of these genes contain HD-binding *cis*-elements in their promoters predominantly with the TTTAATTT sequence, which can be bound by HDG11 *in vitro* and *in vivo*. The coordinated expression of these gene families overlaps fast root elongation. Furthermore, overexpression of *AtEXPA5*, which was dramatically upregulated in *edt1*, resulted in longer primary roots because cells were more extended longitudinally. When combined by crossing the *AtEXPA5*-overexpression lines with one pectin methylesterase inhibitor family protein (PMEI) gene (At5g62360)- or one cellulase (CEL) gene (At2g32990)-overexpression lines, the primary roots of the progeny even exceeded both parents in length. Our results demonstrate that HDG11 directly upregulates cell-wall-loosening protein genes, which is correlated with altered root system architecture, and confirm that cell-wall-loosening proteins play important roles in coordinating cell-wall extensibility with root development. The results of transgene experiments showed that expansin works together with PMEI and CEL to generate synergistic effects on primary root elongation, suggesting that different cell-wall-loosening protein families may function in combination to generate optimal effects on root extensibility.

## Summary

EDT1/HGD11 coordinately upregulates gene families of cell-wall-loosening proteins to alter cell-wall extensibility and promote primary root elongation.

## Introduction

A root system is crucial for plants to absorb water and nutrients from the soil, in addition to anchoring and supporting the plant. A better-shaped root-system architecture would benefit a plant with increased fitness (Aiken and Smucker, 1996; de Dorlodot *et al.*, 2007; Smith and De Smet, 2012). Root development and growth are not only genetically programmed but also constantly influenced by environmental cues. Roots perceive various environmental signals such as water and nutrients, and process these signals to reprogramme their development to adapt to the environment. In this sense, roots have to be very flexible during their development and growth.

It is well established that plant hormones play essential roles in root development and growth (Brunoud *et al.*, 2012; Dello Ioio *et al.*, 2012; Durbak *et al.*, 2012). Among those hormones, auxin seems to play the most important role in root development. It has been found that most hormones that affect the root development seem to act ultimately by modulating auxin activity (Moubayidin *et al.*, 2010; Perilli *et al.*, 2012). Hormones control root development in many ways, one of which is that hormones can change the expression of root cell-wall-loosening protein genes or the activity of cell-wall-loosening proteins (Jan *et al.*, 2004; Liu *et al.*, 2007; Zhao *et al.*, 2012). Regulated cell expansion allows plants to adapt their morphogenesis to survive in different environmental conditions.

Turgor pressure works with cell-wall loosening proteins to regulate cell expansion (Cosgrove, 2005). Cell expansion is driven by turgor pressure created by water uptake and is circumscribed by the extensibility of the cell wall (Brux *et al.*, 2008). However, much less is known about the change in cell walls that must be coordinately loosened during root development for root cell expansion and lateral root emergence.

Plant cells encase themselves within a complex polysaccharide wall, and thus cell expansion must depend on cell-wall loosening through a process of controlled polymer creep. Plant cells loosen their cell walls through molecular modifications of the wall network that result in relaxation of the wall stress. Wall stress relaxation results from scission of a stress-bearing crosslink or from sliding of such a crosslink along a scaffold. Cell-wall enlargement occurs secondarily because of cellular water uptake. Four molecular mechanisms for cell-wall loosening are generally accepted. The four wall-loosening agents are expansins (Cosgrove *et al.*, 2002; Yennawar *et al.*, 2006; Won *et al.*, 2010; ZhiMing *et al.*, 2011), xyloglucan endotransglucosylase/hydrolase (XTHs) (Vissenberg *et al.*, 2003, 2005; Osato *et al.*, 2006; Maris *et al.*, 2009; Tominaga-Wada *et al.*, 2009), endo-(1,4)-β-d-glucanase (Yoshida *et al.*, 2006; Zhu *et al.*, 2006; Yang *et al.*, 2011) and hydroxyl radical (•OH) (Schopfer *et al.*, 2002; Liszkay *et al.*, 2003; Muller *et al.*, 2009).

Expansins are pH-dependent wall-loosening proteins that are first identified from ‘acid growth’. They are involved in cell enlargement and in a variety of developmental processes requiring cell-wall modifications. Expansins are a superfamily that contains four families: α-expansin (EXPA), β-expansin (EXPB), expansin-like A (EXLA) and expansin-like B (EXLB).The polyphyletic group of non-plant expansins, such as those in *Dictyostelium*, can be referred to as expansin-like family X (EXLX). EXPA and EXPB proteins have been demonstrated experimentally to cause cell-wall loosening (Cho and Kende, 1997; Guo *et al.*, 2011), whereas EXLAs and EXLBs proteins are known only from their gene sequences.

XTHs are enzymes involved in the modification of load-bearing cell-wall components. They are also encoded by large multigene families (Maris *et al.*, 2009; Yokoyama *et al.*, 2010). A comprehensive expression analysis of all 33 members of the *Arabidopsis* XTH gene family revealed their tissue specificity and distinct response to hormonal stimulation. Among them, at least 10 genes were expressed predominantly in roots (Yokoyama and Nishitani, 2001).

Cellulose is the major component of the plant cell wall. Besides XTH, plants have a family of secreted endo-(1,4)-β-d-glucanases (also called ‘cellulases’), which belong to glycoside hydrolase family 9 (Henrissat, 1991). In *Arabidopsis*, there are 25 members in this family, and three of them are membrane-bound endoglucanases that are involved in cellulose formation (Cosgrove, 2005). The remaining 22 members are secreted enzymes, mostly of unknown function. The role of endo-(1,4)-β-d-glucanases in wall loosening merits greater attention.

A hydroxyl radical (•OH) is a highly active form of reactive oxygen species and has important roles in signalling and cell death. It was reported that •OH produced in the cell wall can loosen it through destroying the hydrogen bonds by non-enzymatically removing a hydrogen atom from polysaccharides (Fry, 1998; Schopfer, 2001; Lionetti *et al.*, 2007).

Besides cellulose, pectin is one of the main components of the plant cell wall. Pectin-related enzymes have important functions in cell-wall loosening such as pectate lyase, pectin esterase/pectin methylesterase (PME), pectin methylesterase inhibitor family protein (PMEI), and pectinase. Pectin is secreted in a highly methyl-esterified form and is dimethyl-esterified by PME, whose activity leads to stiffer pectin gels and reduced cell growth (Derbyshire *et al.*, 2007; Siedlecka *et al.*, 2008). This is probably one of multiple mechanisms for cell-wall stiffening. Regulation of PME activity by specific PMEIs can therefore play a positive role in cell growth. Overexpression of PMEIs results in root length increases (Lionetti *et al.*, 2007). However, opposite results have also been reported (Peaucelle *et al.*, 2011; Hongo *et al.*, 2012). PME and PMEI may have diverse roles in regulating the ratio of methyl-pectin and demethyl-pectin to affect cell-wall extensibility.

The previously reported gain-of-function *Arabidopsis* mutant *edt1* has enhanced drought tolerance with a more extensive root system than the wild-type plant (Yu *et al.*, 2008). In *edt1* mutant, an HD-ZIP IV transcription factor, HDG11, is activated in most tissues. When overexpressed in rice, *AtEDT1/HDG11* also confers drought tolerance and an improved root system (Yu *et al.*, 2013). To uncover the molecular mechanisms underlying the improved root system architecture of *edt1*, we compared the root transcriptome between the *edt1* mutant and the Columbia (Col-0) wild type at different root development stages using microarray analysis. It was found that cell-wall-related genes, most of which function in loosening the cell wall to facilitate its growth, were significantly upregulated in *edt1*, including expansins, XTHs, pectin-related enzymes, and cellulases. Using yeast-one-hybrid (Y1H) and chromatin immunoprecipitation (ChIP) assays, we further demonstrated that most of the upregulated cell-wall-loosening protein genes were directly regulated by HDG11 at the transcriptional level. Our results suggest that the cell-wall-loosening proteins function coordinately to loosen the cell wall during root development. The regulated expression of cell-wall-loosening protein genes is an indispensable component of root development and growth.

## Materials and methods

### Plant materials and growth conditions

The *Arabidopsis thaliana* Col-0 ecotype was used through this study. The *edt1* mutant was identified previously in the same laboratory (Yu *et al.*, 2008). Seeds were surface sterilized in 10% bleach for 10min and then rinsed five times in sterile water. To overcome dormancy, we imbibed seeds at 4 °C for 2–4 d. The seeds were then germinated and grown on Murashige and Skoog (MS) medium at 22 °C under 16h light/8h dark cycles.

### Measurement of cell length of the root elongation zone

Five-day old seedling roots of wild-type and *EXPA5*-overexpression lines were observed under an Olympus light microscope and photographed. We used the measurement software RULER to measure the cell length of the elongation zone, for three cell columns for each root.

### Microarrays

The seeds were germinated on MS solid medium with or without 50mg l^–1^ of glufosinate ammonium horizontally and the seedlings were then transferred to new vertical MS solid medium. After growing for 3, 6, 10, 15, and 20 d, the roots of *edt1* and the wild type were collected and RNA was extracted using TRIzol reagent (Invitrogen). An Affymetrix gene chip was performed and analysed by Capitolbio Corporation.

### Real-time reverse transcription (RT)-PCR

Total RNA was extracted using TRIzol reagent. First-strand cDNA was synthesized from 1 μg of total RNA in a 20 μl reaction mixture using a Prime Script RT regent kit (Takara). The transcript levels of *EXPA5* were examined using the specific primers 5′-ttagtaatctcgcttctcgtggttc-3′ and 5′-ccataaccttggctatacagattgc-3′. The transcript levels of *EXPB3* were examined using the specific primers 5′-atgcagctttttccagtcatgttag-3′ and 5′-tcacatccaccaacgtaccgtaa-3′. *Arabidopsis UBQ5* was used as an internal control using the specific primers 5′-agaagatcaagcacaagcat-3′ and 5′-cagatcaagcttcaactcct-3′. The PCR was performed on an ABI7000 using the following conditions: 95 °C for 3min, followed by 40 cycles of 95 °C for 20 s, 62 °C for 20 s, and 72 °C for 31 s.

### Semi-quantitative RT-PCR

Total RNA was extracted using TRIzol reagent from 20-d-old roots of the wild type and *edt1*. First-strand cDNA was synthesized from 1 μg of total RNA in a 20 μl reaction mixture using a Prime Script RT regent kit (Takara). The transcript levels of AT1g48930, AT5g62360, AT2g36870, At2g32990, At5g57560, At1g67750, and At1g54970 were examined using the specific primers listed in Supplementary Table S3 at *JXB* online.

### Y1H assay

A Y1H assay was performed as described previously with minor modifications (Wang *et al.*, 2007). A cDNA fragment encoding HDG11 was amplified with the primer pair 5′-ccgctcgagatgagtttcgtcgtcggcgt-3′ and 5′-gctctagaagctgtagttgaagctgtag-3′, and inserted into pAD-GAL4-2.1 to generate the pAD/HDG11 plasmid, and this plasmid was used to produce protein (translationally fused to the GAL4 AD domain) for DNA binding in the Y1H assay. Three copies of the *cis*-DNA elements, containing *Sac*I and *Mlu*I adaptors, were annealed and cloned into the *Sac*I and *Mlu*I sites of the reporter plasmid, pHIS2, which also contained the nutritional reporter gene, *HIS3*. The constructs were confirmed by sequencing.

The pAD/HDG11 construct and the reporter pHIS2 containing the *cis*-DNA element were co-transfected into yeast cells Y187. For the negative control, the pAD/HDG11 vector and the pHIS2 empty plasmid were co-transfected into Y187 yeast cells. Yeast was grown in SD/–Trp–Leu medium and then spotted on SD/–Trp–Leu–His medium in the presence of 10mM 3-aminotriazole (Sigma) at different dilutions. The plates were incubated at 30 ℃ for 4 d and the extent of yeast growth was determined. Normal growth of the cells on the SD/–Trp–Leu–His medium in the presence of 10mM 3-aminotriazole indicated that binding of the transcription factor to the corresponding *cis*-DNA element had occurred.

### Constructs and generation of transgenic plants

To get *35S:: EXPA5* transgenic lines, we isolated the *EXPA5* cDNA from wild-type Col-0 by RT-PCR with forward primer 5′-gtacaaaaaagcaggctatgggagttttagtaatctcgct-3′ and reverse primer 5′-gtacaagaaagctgggtttaataccgaaactgccctcc-3′, cloned it into pDONR207, and subsequently shuttled it into the expression binary vector pCB2004 (Lei *et al.*, 2007).

To get *35S::*At5g62360 (PMEI) transgenic lines, we isolated the At5g62360 cDNA from wild-type Col-0 by RT-PCR with forward primer 5′-gtttgtacaaaaaagcaggctatgggtgaatcttttagattat-3′ and reverse primer 5′-ctttgtacaagaaagctgggtttagccatgaatagaagcaaag-3′, cloned it into pDONR207, and subsequently shuttled it into the expression binary vector pCB2004.

To get *35S::*At2g32990 [cellulase (CEL)] transgenic lines, we isolated the At2g32990 cDNA from wild-type Col-0 by RT-PCR with forward primer 5′-gtttgtacaaaaaagcaggctatgactgtgatgaatcaccgac-3′ and reverse primer 5′-ctttgtacaagaaagctgggtctatctcttataagttgcaacc-3′, cloned it into pDONR207, and subsequently shuttled it into the expression binary vector pCB2004.

For ChIP assays, the *35S::HA-Tag-HDG11* construct was made by primers with a haemagglutinin (HA) tag: 5′-gtttgtacaaaaaagcaggctatgtacccatacgatgttccagattacgctatgagtttcgtcgtcggcgtcg-3′ and 5′-ctttgtacaagaaagctgggttcaagctgtagttgaagctgta-3′, cloned it into pDONR207, and subsequently shuttled it into the expression binary vector pCB2004.

The constructs were introduced into *Agrobacterium tumefaciens* C58C1, which was used to transform the Col-0 wild-type plants as described previously (Clough and Bent, 1998).

### ChIP assay

A ChIP assay was performed as described previously (Lee *et al.*, 2007) using an HA tag-specific monoclonal antibody (Cali-Bio) for immunoprecipitation. Approximately 0.5g of 10-d-old roots was used in each ChIP experiment. The immunoprecipitated chromatin was extracted with phenol/chloroform and the DNA was precipitated with ethanol. ChIP-PCR was then used to verify each promoter segment of related genes using the primers listed in Supplementary Table S4 at *JXB* online.

## Results

### A large number of cell-wall-loosening protein genes are upregulated in *edt1* roots

**Fig. 1.**
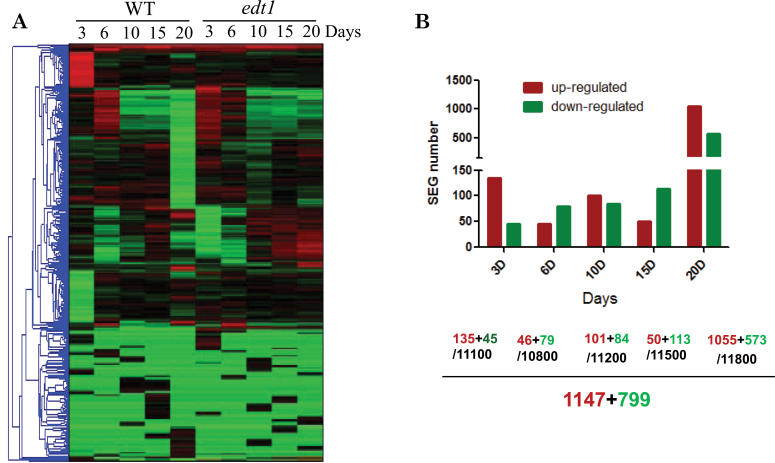
Microarray analysis of *edt1* roots. RNA samples isolated from 3-, 6-, 10-, 15-, and 20-d-old roots of *edt1* and wild-type plants were analysed by microarray. (A) Overview of gene clusters. (B) SEG numbers at different growth stages. Upregulated and downregulated SEG numbers are indicated, together with the total number of genes identified at each specific stage. (This figure is available in colour at *JXB* online.)

The *edt1* mutant is a gain-of-function mutant with enhanced drought tolerance and a well-developed root system (Yu *et al.*, 2008). To gain a view of global expression patterns in the root of the *edt1* mutant, we compared the root transcriptome between *edt1* and the wild type (Col-0). The transcription profiling results showed that there were dramatic changes in expression profiles in *edt1* compared with the wild type (Fig. 1A). In our analysis, the genes were defined as significantly expressed genes (SEGs) if their change was greater than or equal to 2-fold that of the wild type. At different developmental stages, there were different numbers of SEGs. The stage of 20 d had the largest number of SEGs. In total, there were 1147 upregulated SEGs and 799 downregulated SEGs (Fig. 1B).

**Fig. 2.**
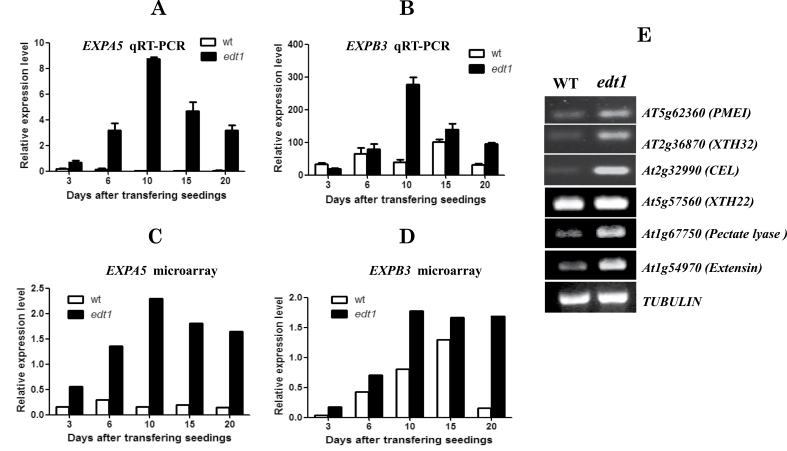
Cell-wall loosening-related genes are coordinately upregulated in *edt1*. (A–D) RNA samples isolated from 3-, 6-, 10-, 15-, and 20-d-old roots of *edt1* and wild-type plants were analysed by real-time RT-PCR using specific primers and a microarray. Results are shown as the relative expression level of *EXPA5* (A) and *EXPB3* (B) by real-time RT-PCR, and the relative expression level of *EXPA5* (C) and *EXPB3* (D) by microarray. (E) Expression levels of other cell-wall-loosening protein genes by RT-PCR. RNA samples isolated from 20-d-old roots of *edt1* and wild-type plants were analysed by semi-quantitative RT-PCR using specific primers.

Gene Ontology (GO) term enrichment analysis of upregulated SEGs showed that cell-wall subcellular localization genes were significantly enriched (Table 1). Moreover, when we looked into the cell-wall-related genes, we found that most of the 72 cell-wall-related genes had functions in cell-wall loosening (Supplementary Table S1 at *JXB* online), such as expansins, XTHs, pectin-related enzymes, glycosyl hydrolases, and extensins. Considering that there are many gene products that do not localize on cell wall but that have functions in cell-wall loosening, we searched all 1147 upregulated genes and found 11 expansins, 13 XTHs, 20 pectin-related enzymes (six pectate lyase lyases, six pectin esterases, five PMEIs, and three pectinases), 29 glycosyl hydrolases, and 24 extensins (Supplementary Table S2 at *JXB* online). These kinds of gene all play key roles in cell-wall loosening, especially expansins and XTHs. *EXPA5* was the most notably changed gene; it had almost no expression in wild-type roots but very high expression in *edt1* roots (Fig. 2A, C, and Supplementary Fig. S1A at *JXB* online). *EXPB3*, expressed mainly in roots of the wild type with reference to the compiled microarray data (Supplementary Fig. S1B), was also significantly upregulated in *edt1* roots (Fig. 2B, D). The upregulation of a representative member for other cell-wall-loosening protein families was also confirmed by semi-quantitative RT-PCR (Fig. 2E). Our data revealed that many families of cell-wall-loosening protein genes are upregulated significantly in *edt1* roots, which may facilitate root growth and development.

**Table 1.** GO term enrichment analysis of SEGs in edt1 rootsGO analysis was performed on the MIPS website (http://mips.helmholtz-muenchen.de/proj/funcatDB/). The lower the *P* value, the higher the degree of enrichment. The top four enrichment functional categories were chosen and are listed in this table. ‘Abs set’ means the gene number in this functional category out of input and ‘Rel set’ means its percentage of the input gene number (which in this table is 1147). ‘Abs genome’ means the gene number in this functional category out of the *Arabidopsis* genome and ‘Rel genome’ means its percentage of the *Arabidopsis* genome.

Functional category	Abs set	Rel set	Abs genome	Rel genome	*P* value
Cell wall	72	6.28	431	1.51	6.79E–25
Oxidative stress response	39	3.4	200	0.7	2.14E–16
Oxygen and radical detoxification	40	3.49	261	0.91	3.88E–13
Osmosensing and response	32	2.79	208	0.73	7.78E–11

### HD-binding *cis-*elements are highly enriched in the promoters of cell-wall-loosening protein genes

As AtHDG11 is a transcription factor belonging to the HD-ZIP IV subfamily, which can bind specific HD-binding *cis-*elements to regulate gene expression, we analysed the promoters of these upregulated SEGs and found that 523 out of the 1147 genes contained HD-binding *cis-*elements in their promoters. The result of GO term enrichment analysis of the 523 genes is shown in Table 2. The cell-wall-related genes were still the most enriched functional category. The ratios of genes with an HD-binding *cis*-element in different functional categories were all significantly higher than the average for the *Arabidopsis* genome (Table 3), which indicates that the upregulated SEGs could be directly regulated by HDG11. Moreover, among the upregulated cell-wall-loosening protein genes, eight of the 11 expansins, six of the 12 XTHs, 14 of the 20 pectin-related enzymes, 18 of the 29 glycosyl hydrolases, and 14 of the 24 extensins contained an HD-binding *cis*-element in their promoters (Supplementary Table S2). There were several types of HD-binding *cis*-element in the upregulated cell-wall loosening protein genes promoters; however, most of them contained AAATTAAA (Table 4). Thus, the AAATTAAA motif appeared to be the predominant HD-binding *cis*-element.

**Table 2.** GO term enrichment analysis of SEGs containing HD-binding cis-element in edt1 rootsSee Table 1 legend for abbreviations.

Functional category	Abs Set	Rel set	Abs genome	Rel genome	*P* value
Cell wall	31	5.92	431	1.51	1.42E–10
Oxidative stress response	19	3.63	200	0.7	6.45E–09
Osmosensing and response	17	3.25	208	0.73	3.57E–07
Oxygen and radical detoxification	16	3.05	261	0.91	3.07E–05

**Table 3.** The ratio of genes with HD-binding cis-element in different functional categories

Functional category	Gene no.	Gene no. with HD-binding *cis*-element	Ratio
Cell wall	72	31	43.1%
Oxidative stress response	39	19	48.7%
Oxygen and radical detoxification	40	17	42.5%
Osmosensing and response	32	16	50.0%
*Arabidopsis* genome	29110	10010	34.4%

**Table 4.** The HD-binding cis-element in the promoters of cell-wall loosening protein genes upregulated in edt1 roots (including the genes coding for proteins that do not localize in the wall)

Gene family	Locus	HD-binding *cis*-element
Expansin	At3g29030	aaattaaa
	At4g28250	aaattaaa
	At2g03090	aaattaaa
	At1g20190	aaattaaa ×2
	At2g20750	aaattaaa ×2
	At2g39700	aaattaaa ×3
	At3g45970	aaattaaa, aaattagt, actaattt
	At1g12560^*a*^	aaattagt ×2, actaattt ×2
XTH	At2g06850	aaattaaa ×4
	At2g36870	aaattaaa ×3
	At4g03210	aaattaaa
	At4g30270	aaattaaa ×3
	At4g37800	aaattaaa ×3
	At5g57560	aaattaaa, aaattagt, actaattt
Pectate lyase	At1g67750	aaattaaa ×3
	At4g24780	aaattaaa ×2
	At1g04680	aaattaaa, aaattagt, actaattt
	At3g24670^*a*^	aaattagt ×2, actaattt ×2
Pectinesterase	At2g45220	aaattaaa ×2, aaattagt, actaattt
	At1g02810	aaattaaa
	At5g47500	aaattaaa, aaattagt, actaattt
	At3g10720	aaattaaa
Pectinase	At1g65570^*a*^	actaattt ×2, aaattagt ×2
	At5g14650	aaattaaa ×2, aaattagt, actaattt
	At3g07970	aaattaaa, aaattagt, actaattt
PMEI	At5g62350	aaattaaa ×3, aaattagt, actaattt
	At1g62770	aaattaaa
	At5g62360	aaattaaa ×2
Glycosyl hydrolase	At3g60130	aaattaaa
	At1g51470	aaattaaa
	At1g47600	aaattaaa
	At3g07320	aaattaaa
	At4g18340	aaattaaa
	At5g63800	aaattaaa
	At5g34940	aaattaaa
	At5g15870	aaattaaa
	At4g02290	aaattaaa
	At4g19810	aaattaaa ×2
	At2g32990	aaattaaa ×2, aaattagt ×2, actaattt ×2
	At3g28180	aaattaaa ×3, aaattagt ×2, actaattt ×2
	At1g60140	aaattaaa ×3, aaattagt, actaattt
	At3g60140	aaattaaa ×4, aaattagt ×2, actaattt ×2
	At1g61820	aaattaaa ×5
	At1g02640	aaattaaa ×5
	At2g27500^*a*^	aaattagt
	At5g49360^*a*^	aaattagt, actaattt
Extensin	At4g38770	aaattaaa
	At4g02270	aaattaaa
	At5g05500	aaattaaa, aaattagt, actaattt
	At3g54580	aaattaaa ×2
	At5g49080	aaattaaa
	At4g08410	aaattaaa, aaattagt, actaattt
	At1g21310	aaattaaa
	At5g06630	aaattaaa
	At3g28550	aaattaaa ×2, aaattagt ×2, actaattt ×2
	At3g06750	aaattaaa
	At1g76930	aaattaaa ×2
	At5g49280	aaattaaa
	At5g09480	aaattaaa
	At2g43150	aaattaaa

^*a*^ Genes whose promoter does not contain the ‘aaattaaa’ motif.

### HDG11 can directly regulate cell-wall-loosening protein gene expression

**Fig. 3.**
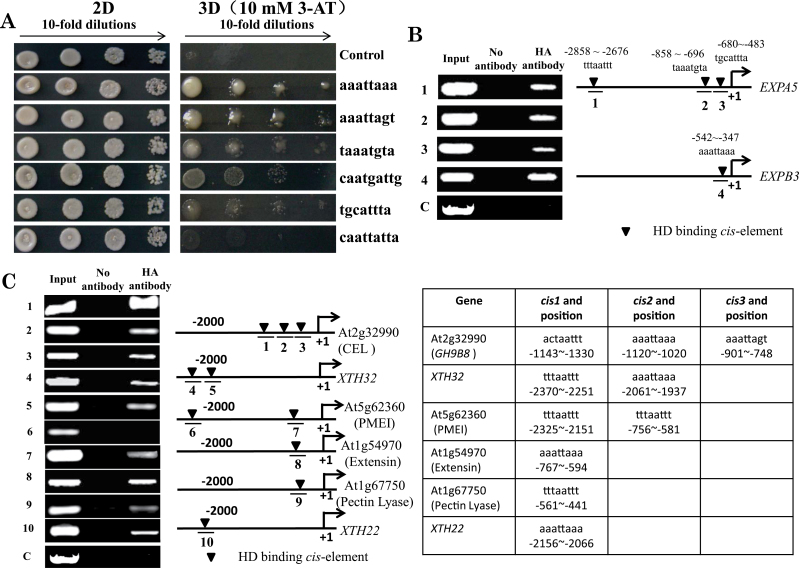
HDG11 binds to the promoters in Y1H and ChIP assays. (A) Y1H assay in yeast strain Y187. The two vectors pGADT7/*HDG11* and pHIS2/*cis*-element were transformed into Y187, and pGADT7/*HDG11* and empty pHIS2 were used as negative controls. (B) ChIP-PCR for *EXPA5* and *EXPB3* promoters. The predicted HD-binding elements are indicated with inverted triangles, above which their sequence and position are shown. 1, *EXPA5* promoter –2858 to –2676bp containing tttaattt; 2, *EXPA5* promoter –858 to –696bp containing taaatgta; 3, EXPA5 promoter –680 to –483bp containing tgcattta; 4, *EXPB3* promoter –542 to –347bp containing aaattaaa; C, negative control using *TUB8* promoter. (C) ChIP-PCR for the *GH9B8*, *XTH32*, PMEI, extensin, pectin lyase, and *XTH22* promoters. The predicted HD-binding elements and their sequence and position are listed in the table on the right. 1, *GH9B8* promoter –1143 to –1330bp containing actaattt; 2, *GH9B8* promoter –1120 to –1020bp containing aaattaaa; 3, *GH9B8* promoter –901 to –748bp containing aaattagt; 4, *XTH32* promoter –2370 to –2251 containing bp tttaattt; 5, *XTH32* promoter –2061 to –1937bp containing aaattaaa; 6, PMEI promoter –2325 to –2151bp containing tttaattt; 7, PMEI promoter –756 to –581bp containing tttaattt and aaattaaa; 8, extensin promoter –767 to –594bp containing aaattaaa; 9, pectin lyase promoter –561 to –441bp containing tttaattt; 10, *XTH22* promoter –2156 to –2066bp containing aaattaaa; C, negative control using the *TUB8* promoter.

HD-ZIP IV transcription factors show a binding preference for variant HD-binding sequences. *HDG7*, *HDG9*, *ATML1*, and *PDF2* recombinant proteins bind to the GCATT(A/T)AATGC consensus sequence, which overlaps with the L1 box sequence TAAATG(C/T)A recognized *in vitro* by ATML1 and GL2 (Ariel *et al.*, 2007; Tominaga-Wada *et al.*, 2009). According to the *Arabidopsis* Gene Regulatory Information Server (AGRIS), we chose six sequences, listed in Fig. 3A, as potential HDG11-binding sites in a Y1H assay. HDG11 showed different binding affinities to these sequences. The sequence aaattaaa was the favourite sequence, following by aaattagt, taaatgta (the L1 box), caatgattg, and tgcattta. HDG11 failed to bind caattatta (Fig. 3A). Most of the upregulated cell-wall-loosening protein genes contained aaattaaa sequence in their promoters (Table 4), indicating the possibility of HDG11 transcriptionally regulating these cell-wall-loosening protein genes.

In the *EXPA5* promoter, there are three potential HDG11-binding sites, all of which are predicted to be bound by HDG11. In *the EXPB3* promoter, there is one potential HDG11-binding site. To confirm this, we generated HA-tagged HDG11-overexpressing lines for a ChIP assay to test whether HDG11 could bind to the promoters of *EXPA5* and *EXPB3 in vivo*. The ChIP-PCR results showed that HDG11 was able to bind all three sites in the *EXPA5* promoter and the one site in the *EXPB3* promoter (Fig. 3B), consistent with the Y1H results. We also tested the binding of HDG11 to the promoters of other cell-wall-loosening protein genes *in vivo*. The results in Fig. 3C showed that HDG11 could bind to most of the predicted HD-binding *cis*-elements in the promoter of the upregulated cell-wall-loosening genes. These results demonstrated that HDG11 can directly bind to the promoters of the cell-wall-loosening protein genes and potentially activate their expression to coordinate cell-wall extensibility with root development.

### Overexpression of *EXPA5* stimulates root cell elongation

**Fig. 4.**
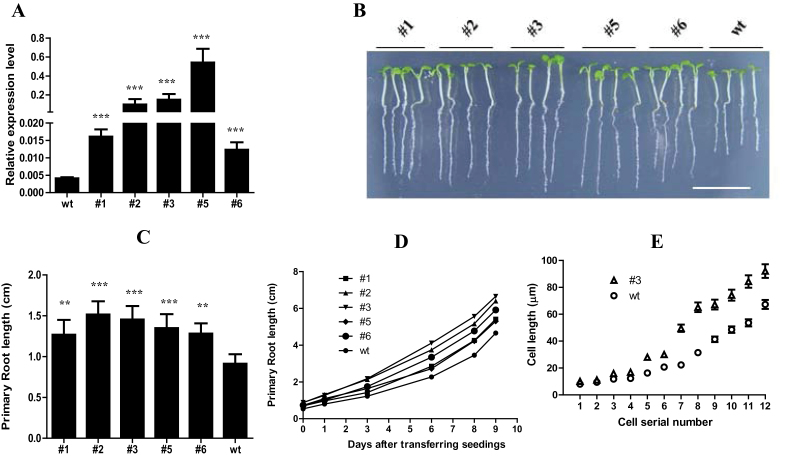
Root architecture of *EXPA5-*overexpression transgenic plants. (A) Relative expression level of *EXPA5* in five transgenic lines. RNA samples isolated from 10-d-old roots of mutants and wild type were analysed by real-time RT-PCR using specific primers. Values are means±standard deviation (SD) (*n*=3). Statistically significant differences are indicated (***P<0.001). (B) The primary root of 5-d-old mutant seedlings (*35S::EXPA5*) was longer than that of the wild-type seedlings of the same age on MS medium. Bar, 1cm. (C) The primary root length of plants shown in (B). Presented are means±SD (*n*=3, >50 seeds per replicate experiment). Statistically significant differences are indicated (**P<0.05; ***P<0.001). (D) Primary root elongation curve of the wild-type and mutant seedlings grown on MS medium. (E) Length of cells from the elongation zone of the wild type and one of the transgenic lines. (This figure is available in colour at *JXB* online.)

Among the upregulated cell-wall-loosening protein genes, *EXPA5* was the most notable because its expression level was barely detectable in wild-type roots but was very high in *edt1* roots (Fig. 2A and Supplementary Fig. S1A). We hypothesized that constitutively overexpressing this gene driven by the 35S promoter in the wild-type plant might affect the root growth. Five transgenic lines were obtained and their *EXPA5* expression levels in roots are shown in Fig. 4A. The primary roots of these lines were significantly longer than that of the wild type (Fig. 4B, C). The root growth curves showed that primary root elongation of the transgenic lines was faster than that of the wild type from d 1 to d 9 in this experiment (Fig. 4D). The increased elongation was caused by increased cell length rather than cell number (Fig. 4E and Supplementary Fig. S2 at *JXB* online). Thus, altering the expression of a single expansin gene brought about a marked change in root length, which suggests that expansin proteins have a significant impact on cell-wall loosening and elasticity that are important during root development.

### Combined overexpression of *EXPA5* with either PMEI or CEL generates a synergistic effect on primary root elongation, while overexpression of a PMEI or CEL gene alone does not

Having demonstrated that overexpression of *EXPA5* could make primary roots elongate faster, we wondered if overexpression of other cell-wall-loosening protein genes could also have similar effects. Therefore, we tested one CEL gene (At2g32990) and one PMEI gene (At5g62360) and found that neither transgenic line had a primary root longer than the wild-type control (Supplementary Fig. S3 at *JXB* online). These results suggested that some of the cell-wall-loosening proteins may not function alone in root development and that some coordination among those proteins is probably required for their optimal function.

**Fig. 5.**
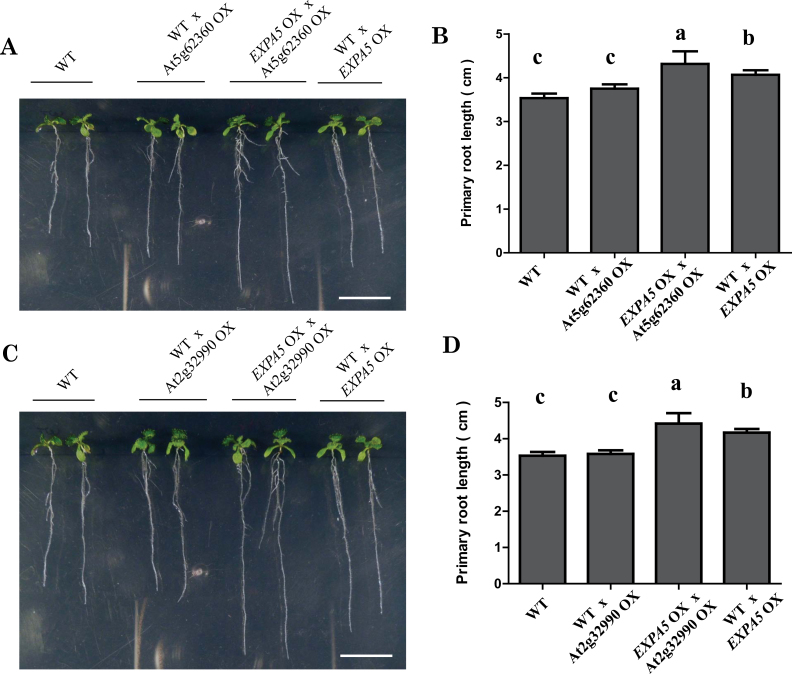
Root architecture of PMEI- and *EXPA5-* or CEL- and *EXPA5-*overexpression transgenic plants. (A)The primary root of 8-d-old wild-type seedlings and F1 progeny of the cross between the wild type (♀)×PMEI-overexpressing line (OX) (♂), *EXPA5* OX (♀)×PMEI OX, and the wild type (♀)×*EXPA5* OX (♂) on MS medium. (B) The primary root length of plants shown in (A). Presented are means±SD (*n*=3, >50 seeds per replicate experiment). Different letters indicate significant differences using SPSS analysis. (C)The primary root of 8-d-old wild-type seedlings and F1 progeny of the cross between the wild type (♀)×CEL OX (♂), *EXPA5* OX (♀)×CEL OX, and wild type (♀)×*EXPA5* OX (♂) on MS medium. (D) The primary root length of the plants shown in (C). Presented are means±SD (*n*=3, >50 seeds per replicate experiment). Different letter indicate significant differences using SPSS analysis. (This figure is available in colour at *JXB* online.)

Cell-wall loosening is a strictly controlled progress that needs many proteins to coordinate properly. To test this idea, we crossed the *EXPA5*-overexpressing line with a PMEI- or CLE-overexpressing line. Interestingly, F1 plants overexpressing *EXPA5* plus either PMEI or CLE produced primary roots that were significantly longer than those of either parents (Fig. 5). These results demonstrated that when either PMEI or CLE combined with *EXPA5*, the primary root elongation was promoted and the primary root length exceeded the *EXPA5-*overexpressing line, suggesting that cooperation may exist among the cell-wall-loosening proteins in regulating cell-wall elasticity at least between EXPA5 and PMEI or CLE proteins.

## Discussion

To uncover the molecular mechanisms underlying the well-developed root system of the *edt1* mutant, we compared the root transcript profiles of *edt1* with that of the wild type and found that a series of cell-wall-loosening protein genes were upregulated in *edt1*. A promoter scan of these genes for *cis*-elements revealed that most of the cell-wall-loosening protein genes contained at least one HD-binding *cis-*element in their promoter. The aaattaaa sequence was the dominant *cis*-element (Table 4), and showed high affinity to the HDG11 protein in a Y1H assay (Fig. 3A). By Y1H and ChIP-PCR assays, we showed that HDG11 could bind to the promoter segments with an HD-binding *cis*-element of these cell-wall-loosening protein genes. Based on these results, it is clear that HDG11 can directly regulate cell-wall-loosening protein genes and hence alter cell-wall extensibility of roots, which at least partially contributes to the well-developed root system of *edt1* mutant.

Plant growth and development need to break through the limit of the plant cell wall. The regulation of cell-wall extensibility is a necessary process accompanying cell expansion and division. In fast-growing tissues such as roots, loosening of the cell wall is a well-regulated process and is coordinated with development. Enhanced cell-wall loosening is also a response of roots to water deficit (Hsiao and Xu, 2000; Sharp *et al.*, 2004). Our results with the *edt1* mutant further demonstrate that cell-wall loosening is important to plant development and growth.

The cell-wall-loosening process is regulated by many factors including hormones and environmental cues and needs a set of proteins to cooperate in an orderly and closely regulated (Cosgrove, 2005). The results of our microarray analysis showed that a series of cell-wall-loosening protein genes are upregulated in *edt1* roots, including expansins, XTHs, pectin lyases, cellulases, PMEIs, and extensins, which cover almost all the cell-wall-loosening protein gene families. Interestingly, the cell-wall-loosening protein genes were coordinately upregulated in *edt1* roots, which was made possible by the HD-binding *cis*-elements in their promoters and activated expression of *EDT1/HDG11*. Moreover, the positive role of brassinosteroids in plant growth is performed partly by activating cell-wall-loosening protein genes (Nemhauser *et al.*, 2004; Bai *et al.*, 2012). Such coordinated upregulation may be a general mechanism in root development.

Among the cell-wall loosening protein genes, the expansin gene family has important roles in cell-wall loosening (Cosgrove *et al.*, 2002). For instance, cell-wall modification is required for root-hair growth. The root-hair-specific expansin A family is required for root-hair elongation in rice. Loss of *EXPA17* (*OsEXPA17*) produces short root hairs (ZhiMing *et al.*, 2011). Lateral root formation is a major determinant of the root-system architecture and defines the capability of a plant to acquire water and nutrients from soil. During *Arabidopsis* lateral root emergence, *EXPANSIN14* (*EXP14*) is directly activated by the LATERAL ORGAN BOUNDARIES DOMAIN 18 (LBD18) protein, a transcriptional activator playing important roles in lateral organ development (Lee *et al.*, 2013). In the *edt1* mutant, *EXPA5* was dramatically upregulated in roots. When overexpressed, it improved root elongation (Fig. 4). Nevertheless, when there was overexpression of only the PMEI or CEL gene, root elongation was not significantly affected (Supplementary Fig. S3). However, when *EXAP5* was co-overexpressed with these two cell-wall-loosening genes, the roots grew faster (Fig. 5). Although we did not test all of the cell-wall-loosening genes, from the microarray analysis and our experiment data, we speculate that the process of cell-wall loosening needs many proteins to cooperate closely. It has been reported that overexpression of one PMEI gene can increase root length, such as PMEI-1 (At1g48020) or PMEI-2 (At3g17220) (Lionetti *et al.*, 2007). The PMEI (At5g62360) that we overexpressed has relatively low protein identity to these two PMEIs, which may be why overexpression of At5g62360 did not stimulate root elongation. Perhaps this PMEI needs to cooperate with other cell-wall-loosening proteins to play its role in root growth. If different combinations of cell-wall-loosening proteins generate different cell-wall modifications, this would increase the flexibility of the cell wall to meet the various demands of development in plants.

In conclusion, our study with the *edt1* mutant demonstrates that the homeodomain transcription factor EDT1/HDG11 is able to directly and coordinately upregulate several gene families of cell-wall-loosening proteins, leading to the altered cell-wall extensibility required in root development. In addition, our results suggest that expansin may work in combination with PMEI or CEL to generate synergistic effects on cell-wall extension, which provides a mechanism for plants to adjust their cell-wall physical properties.

## Supplementary data

Supplementary data are available at *JXB* online.

Supplementary Fig. S1. EXPA5 (A) and EXPB3 (B) expression pattern by AtGenExpress Visualization Tool (AVT).

Supplementary Fig. S2. The cells in the elongation zone of the EXPA5-overexpressing line are longer than that of the wild type.

Supplementary Fig. S3. The root architecture of PMEI (At5g62360)- or CEL (At2g32990)-overexpressing transgenic plants.

Supplementary Table S1. The 72 cell-wall related genes upregulated in *edt1* mutant roots.

Supplementary Table S2. The upregulated cell-wall-loosening protein genes with HD-binding *cis*-elements in their promoter and the ratio in each category.

Supplementary Table S3. Primer sequences for semi-quantitative PCR.

Supplementary Table S4. Primer sequences for ChIP-PCR.

Supplementary Data

## Abbreviations:

CEL: cellulase
ChIP: chromatin immunoprecipitation
EXP: expansin
GO: Gene Ontology
MS: Murashige and Skoog
PMEI: pectin methylesterase inhibitor
RT-PCR: reverse transcription-PCR
SD: standard deviation
SEG: significantly expressed gene
XTH: xyloglucan endotransglucosylase/hydrolase
Y1H: yeast one-hybrid.
