# Supplementary material for: HDG11 upregulates cell-wall-loosening protein genes to promote root elongation in Arabidopsis
Source: J Exp Bot. 2014 May 12;65(15):4285–95. doi: 10.1093/jxb/eru202 (PMC4112634; doi:10.1093/jxb/eru202)
Supplement: Supplementary Data [file supp_65_15_4285__index.html]

HDG11 upregulates cell-wall-loosening protein genes to promote root elongation in Arabidopsis — HDG11 upregulates cell-wall-loosening protein genes to promote root elongation in Arabidopsis — Supplementary Data 

# HDG11 upregulates cell-wall-loosening protein genes to promote root elongation in *Arabidopsis*

## Supplementary Data

Data files

**Files in this Data Supplement:**

- Supplementary Data - Supplementary Data
