# Supplementary material for: HDG11 upregulates cell-wall-loosening protein genes to promote root elongation in Arabidopsis
Source: J Exp Bot. 2014 May 12;65(15):4285–95. doi: 10.1093/jxb/eru202 (PMC4112634; doi:10.1093/jxb/eru202)
Supplement: Supplementary Data [file supp_eru202_jexbot122051_file001.pdf]

Supplemental materials for " HDG11 upregulates cell wall-loosening protein genes to promote root elongation in Arabidopsis" by Xu *et al*.

Table S1. The 72 cell-wall related genes up-regulated in *edt1* mutant roots.

Locus ID	Description	*edt1*/WT 3D	*edt1*/WT 6D	*edt1*/WT 10D	*edt1*/WT 15D	*edt1*/WT 20D
At1g02810	pectinesterase	0.94	0.95	1.04	1.02	2.62
At1g04040	acid phosphatase	1.27	1.14	0.79	0.99	2.44
At1g04680	pectate lyase	0.84	0.83	1.11	1.11	2.54
At1g05240	peroxidase	1.67	1.08	0.48	0.73	8.94
At1g12560	expansin,EXPA7	1.34	0.91	1.11	1.10	3.20
At1g20190	expansin,EXPA11	1.04	1.07	1.06	0.82	3.80
At1g21310	extensin-like	1.06	1.31	0.93	1.01	3.36
At1g33590	LRR protein	1.25	0.79	1.03	1.01	3.83
At1g35140	protein disulfide isomerase	1.12	0.94	0.96	1.07	0.85
At1g41830	multi-copper oxidase	1.11	1.01	0.85	0.85	2.55
At1g53840	pectinesterase	0.97	0.94	1.08	0.99	2.65
At1g54970	extensin-like	2.10	0.94	0.71	0.76	5.93
At1g58370	(1,4)-beta-xylan endohydrolase	1.76	0.88	0.57	0.74	2.33
At1g62770	pectin methylesterase inhibitor	1.29	0.91	0.90	1.03	5.03
At1g62980	expansin,EXPA18	1.57	0.98	0.54	0.77	3.67
At1g65310	xyloglucosyl transferase	1.63	1.27	0.69	1.13	5.52
At1g67750	pectate lyase	-	-	-	-	3.72
At1g69530	expansin,EXPA1	0.90	0.68	2.17	0.85	1.55
At1g75750	gibberellin-regulated protein 1	0.77	0.96	0.63	0.84	3.42
At1g75830	PDF1.1	136	-	-	-	-
At1g76930	extensin-like	0.94	1.11	1.77	1.57	2.92
At1g78830	lectin family protein	2.22	1.03	0.79	1.13	7.41
At1g80240	expressed protein	0.82	0.74	0.51	0.65	3.91
At2g03090	expansin,EXPA15	0.95	1.61	2.58	0.89	1.96
At2g06850	xyloglucosyl transferase	0.87	0.87	0.78	0.85	2.54
At2g20750	expansin,EXPB1	1.26	1.20	0.81	1.20	3.52
At2g24980	extensin-like	1.20	1.20	0.89	1.32	2.89
At2g26010	PDF1.3	31.0	-	-	-	-
At2g39700	expansin,EXPA4	1.35	0.90	1.25	0.81	2.24
At2g43150	extensin-like	0.95	1.10	1.10	0.98	2.01

At2g44450	Glycosyl hydrolase	1.01	0.85	1.07	0.93	3.08
At2g45220	pectinesterase	1.47	1.02	0.60	0.95	14.44
At3g07010	pectate lyase	1.09	0.90	0.72	0.64	2.57
At3g10720	pectinesterase	0.99	0.69	0.61	1.05	2.28
At3g16240	delta tonoplast integral protein	1.14	0.62	0.65	0.68	5.72
At3g24670	pectate lyase	1.41	1.00	0.49	0.87	2.96
At3g28550	extensin-like	0.93	1.23	0.84	1.36	2.94
At3g29030	expansin,EXPA5	3.47	4.69	14.93	9.30	10.97
At3g45960	expansin family protein (EXPL3)	0.65	0.81	1.62	0.97	6.35
At3g45970	expansin family protein (EXPL1)	0.87	1.00	1.30	1.07	2.64
At3g54580	extensin-like	1.10	1.10	0.81	1.25	4.47
At3g54590	extensin-like	1.16	1.17	0.85	1.16	3.27
At3g62020	germin-like protein (GLP10)	1.79	0.91	0.56	0.83	2.18
At3g62680	extensin-like	1.59	0.93	0.77	0.93	8.68
At4g00080	pectin methylesterase inhibitor	0.95	0.75	0.70	0.84	3.00
At4g08400	extensin-like	1.25	1.19	0.86	1.23	2.22
At4g08410	extensin-like	1.38	0.91	0.72	1.08	3.62
At4g08950	phosphate-responsive protein	0.89	0.72	0.91	0.64	2.90
At4g20830	oxidase precursor	1.36	0.85	0.78	0.85	3.51
At4g24780	pectate lyase	1.17	0.84	1.15	0.74	2.06
At4g28250	beta-expansin,EXPB3	4.37	1.66	2.21	1.28	10.95
At4g30270	MERI-5 protein	0.80	0.58	0.74	0.77	4.10
At4g38770	extensin-like	1.15	1.18	1.87	2.06	5.50
At5g04960	pectinesterase	1.43	0.94	0.77	1.03	8.63
At5g06630	extensin-like	1.16	1.30	0.94	1.18	3.10
At5g06640	extensin-like	1.06	1.22	0.81	1.14	5.13
At5g06860	polygalacturonase inhibiting protein	0.97	0.85	0.94	1.01	4.17
At5g16590	protein kinase	1.38	1.14	0.73	1.05	2.05
At5g17820	peroxidase 57	1.60	1.01	0.62	1.01	5.00
At5g25460	expressed protein	0.92	1.06	0.81	0.76	2.46
At5g42100	glycosyl hydrolase	0.71	0.87	0.96	1.07	1.06
At5g42180	peroxidase 64	1.07	1.18	0.78	1.09	4.56
At5g47500	pectinesterase	0.65	0.59	1.00	0.87	2.62
At5g48900	pectate lyase	0.98	0.85	1.17	0.90	2.85
At5g49080	extensin-like	1.37	1.54	0.89	1.05	4.24
At5g51550	phosphate-responsive 1	1.18	0.56	0.92	1.15	2.23

At5g57560	xyloglucosyl transferase	1.08	0.80	1.14	0.87	4.59
At5g59320	lipid transfer protein	0.53	0.86	2.03	1.21	0.27
At5g62340	pectin methylesterase inhibitor	1.21	0.59	0.84	1.31	2.11
At5g62350	pectin methylesterase inhibitor	1.15	1.15	1.60	1.03	2.06
At5g62360	pectin methylesterase inhibitor	1.15	1.15	1.60	1.03	2.06
At5g64260	phosphate-responsive protein	1.17	0.99	1.02	0.97	8.68

Table S2. The upregulated cell wall loosening protein genes with HD-binding cis elements in their promoter and the ratio in each category.

**Gene family**	**SEG genes and their number**	**With HD-binding *cis*-element**
Expansin	**11** (At2g20750, At4g28250, At3g45970, At3g45960, At1g69530, At1g20190, At2g03090, At1g62980, At2g39700, At3g29030, At1g12560)	**8 (72.7%)** (At2g20750, At4g28250, At3g45970, At1g20190, At2g03090, At2g39700, At3g29030, At1g12560)
Endoxyloglucan transferase (XTH)	**13** (At1g32170, At1g65310, At2g06850, At2g36870, At4g03210, At4g14130, At4g25810, At4g25820, At4g30270, At4g30280, At4g37800, At5g57560, At5g65730)	**6 (46.2%)** (At2g06850, At2g36870, At4g03210, At4g30270, At4g37800, At5g57560)
Pectin related enzymes	**20** (At1g67750, At4g24780, At1g04680, At3g07010, At3g24670, At5g48900; At5g62350, At4g00080, At1g62770, At5g62360, At5g62340; At2g45220, At5g04960, At1g53840, At1g02810, At5g47500, At3g10720 ; At1g65570, At5g14650, At3g07970)	**14 (70%)** (At1g67750, At4g24780, At1g04680, At3g24670 ; At5g62350, At1g62770, At5g62360; At2g45220, At1g02810, At5g47500, At3g10720 ; At1g65570, At5g14650, At3g07970)
Glycosyl hydrolase	**29** (At3g60130, At3g62740, At2g44450, At2g44480, At1g61820, At3g60140, At1g51470, At1g47600, At1g58370, At3g55430, At3g07320, At5g42100, At4g18340, At2g27500, At4g16260, At4g19810, At5g49360, At1g02640, At5g63800, At5g34940,, At5g15870, At4g02290, At2g32990, At5g35740, At3g10740, At3g28180, At1g23480, At1g60140, At5g53990)	**18 (62.1%)** (At3g60130, At1g61820, At3g60140, At1g51470, At1g47600, At3g07320, At4g18340, At2g27500, At4g19810, At5g49360, At1g02640, At5g63800, At5g34940,, At5g15870, At4g02290, At2g32990, At1g23480, At1g60140)
Extensin	**24** (At3g09925, At1g76930, At4g38770, At3g09925, At4g02270, At5g05500, At5g06640, At3g54580,	**14 (58.3%)** (At4g38770, At4g02270, At5g05500, At3g54580, At5g49080,

	At5g49080, At3g49840, At4g08410, At2g33790, At1g21310, At3g54590, At5g06630, At3g28550, At3g06750, At1g76930, At2g24980, At5g49280, At1g28290, At2g47540, At5g09520, At5g09480, At4g08400, At2g43150)	At4g08410, At1g21310, At5g06630, At3g28550, At3g06750, At1g76930, At5g49280, At5g09480, At2g43150)

Table S3. Primer sequences of semi-quantitative PCR.

Name	Sequence
AT5g62360 P1	ATTCAATCATCATCACTTCCTCAC
AT5g62360 P2	TGTGAAAGTGCATGATGATTTTACG
AT2g36870 P1	CAAGCTCCAAGGTTGGTTCCC
AT2g36870 P2	CCTGATCTGAATGGCTTCACTG
AT1g48930 P1	GTTTGGTGGATCATTGTTCGGC
AT1g48930 P2	ATCGTTGAGCCTCAAAGAACAAG
At2g32990 P1	ATGACTGTGATGAATCACCGACA
At2g32990 P2	ATGCTTCACCGTAGTCAAACCC
At1g54970 P1	TCCGGTCTACAAAAAGTCTCCAAG
At1g54970 P2	AACACCACGTGGAAGTATCCCTT
At5g57560 P1	ATGGCGATCACTTACTTGCTTCCT
At5g57560 P2	TGGAGACTTTACCAAACAAGTACTC
At1g67750 P1	ATGAGAATGACACTTGTTCACTTG
At1g67750 P2	GCATCTCCAACAGTCGTCAATTG

Table S4. Primer sequences for ChIP-PCR.

Name	Sequence
EXPA5_CHIP_P1	TAGCGGCATAAATCTTCATGAACC
EXPA5_CHIP_P2	TGAGTACTTGGACCCAACATTGAAT
EXPA5_CHIP_P3	TGTGTTCAAAAACCTTCAACATC
EXPA5_CHIP_P4	GAAGAACGGTTGGGACTTTTCATA
EXPA5_CHIP_P5	CGAATACAATGTTGCTCCAATTTC

EXPA5_CHIP_P6	GTTTGAGTGTTTGAGACAGACAAT
EXPB3_CHIP_P1	ACCGAAATTGAAGAAAAGCTTGTC
EXPB3_CHIP_P2	CAGAAAATTCGAATCTCACACCAA
At2g32990_ChIP_P1	AGAATATCGAGTAGGATTTTTAATG
At2g32990_ChIP_P2	CGTTTATACACTTTCATTATACTGT
At2g32990_ChIP_P3	AATAGTTAGGCCCACTTTGAAAATG
At2g32990_ChIP_P4	TTGGCAGTAATTCAGGTTTCCG
At2g32990_ChIP_P5	ACCCATTTAGATCCATCCTATAT
At2g32990_ChIP_P6	GCTGGGTTCACTGTTACAAATTAT
At5g62360_ChIP_P1	ATGTTTGACAATTCTCTTTCACACC
At5g62360_ChIP_P2	TGTCACCGTTTTCAAGTAGTTATGT
At5g62360_ChIP_P3	GGTCGACATCTTTTAATCTTTAGTG
At5g62360_ChIP_P4	ACAATCGCAAATATGAAACCCAAA
At2g36870_ChIP_P1	ATTTCAGCATCGTCCTTCCGGG
At2g36870_ChIP_P2	CCGAACCTCTTTTGTCTCACGG
At2g36870_ChIP_P3	AGTTTATTGAGTTAAGTGGATTCCG
At2g36870_ChIP_P4	CGGCATATGTATTGCTTACTAGTTA
At1g54970_ChIP_P1	TGAATGCACATGTTTAAAGATTCGG
At1g54970_ChIP_P2	GGAGGCCACATAAAGTCCAATATA
At5g57560_ChIP_P1	CTTGTTCATGGGCTATCATCTC
At5g57560_ChIP_P2	AGGGTTCGACACCTGACAAGTT
At1g67750_ChIP_P1	TTTAGCGGGAAAACGGAATTTTAC
At1g67750_ChIP_P2	ACAAATAGACTTAAATCTTATTTTCC

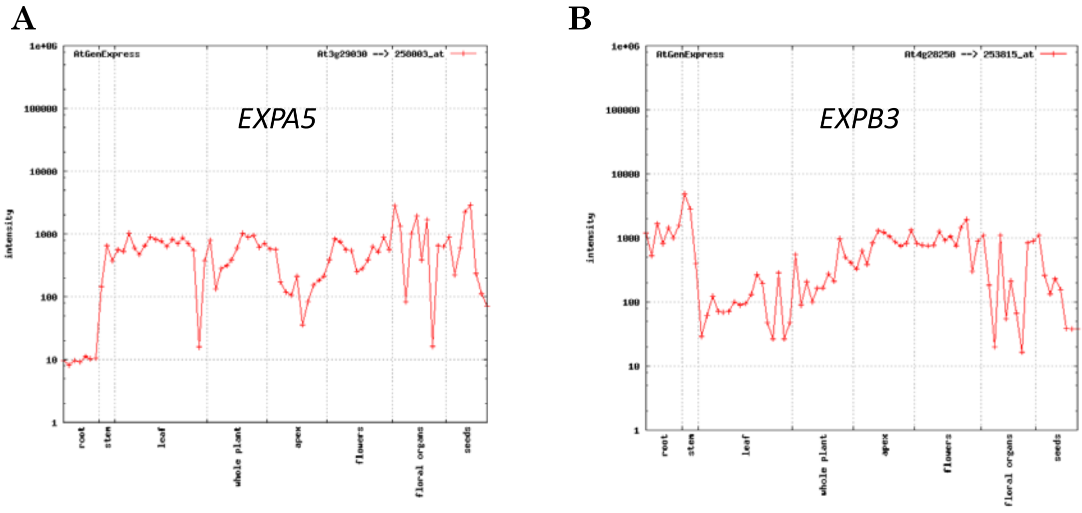

Figure S1. ***EXPA5*** **(A) and** ***EXPB3*** **(B) expression pattern by AtGenExpress Visualization Tool (AVT).**

WT *EXPA5* OX

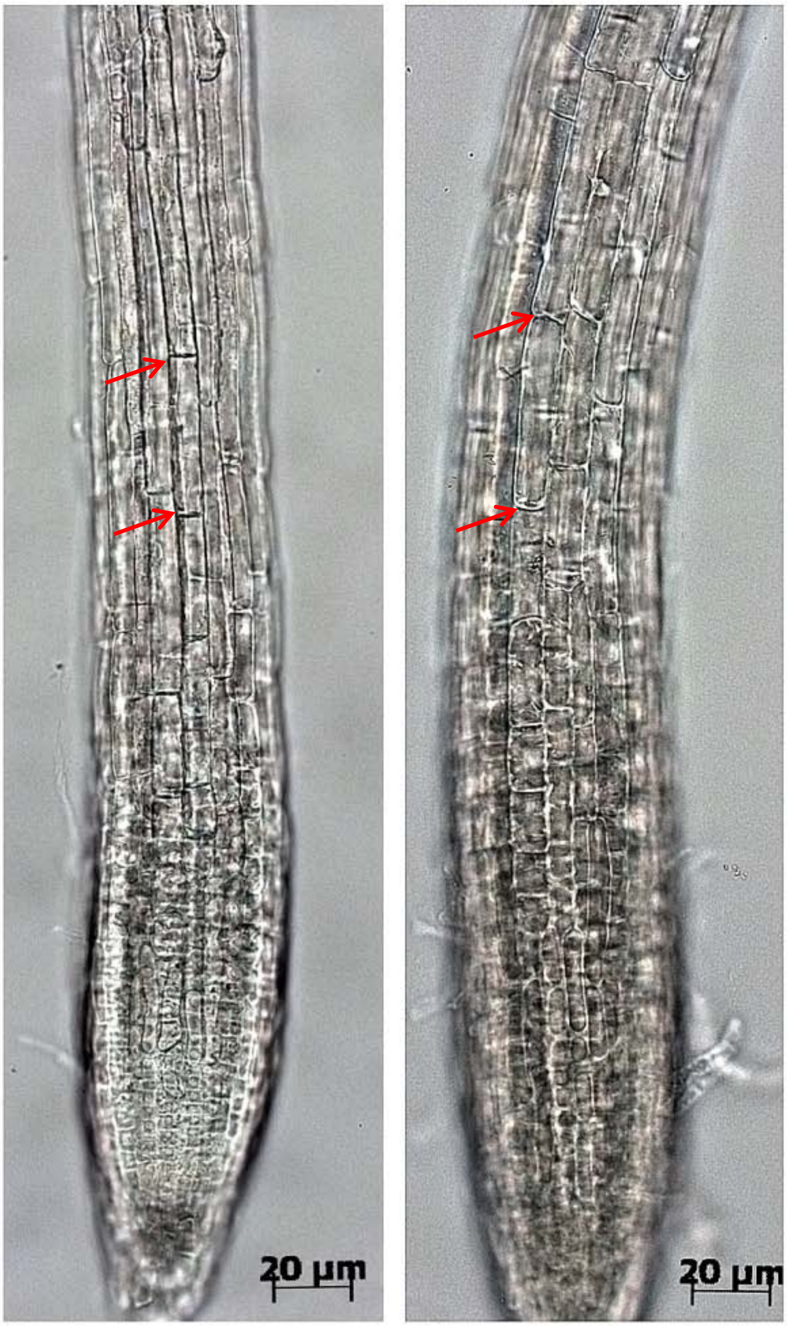

Figure S2. The cells in elongation zone of *EXPA5*-overexpressing line is longer than that of the wild type. The cell between two arrows is the fifth cell of elongation zone.

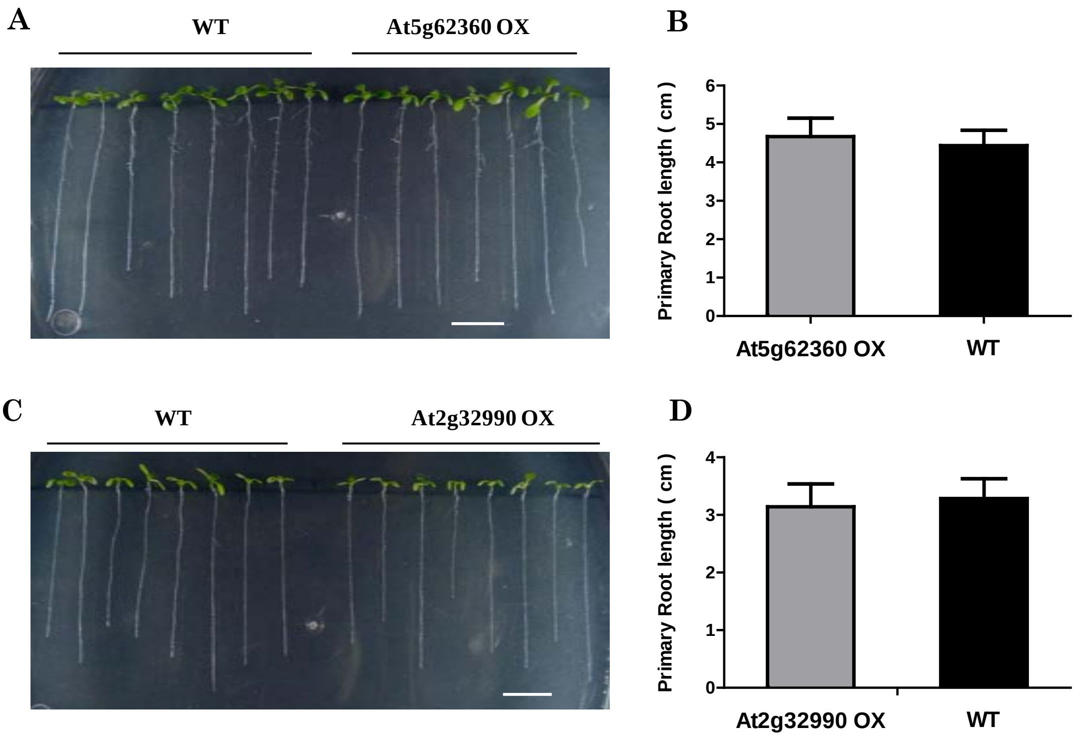

Figure S3. The root architecture of PMEI (At5g62360) or CEL (At2g32990) overexpression transgenic plants.

A. The primary root of 8-d-old wild type and PMEI over-expression transgenic plants on MS medium.

B. The primary root length of plants shown in (A). Presented are means ± SD (n = 3, >50 seeds per replicate experiment). There was no significant difference between wild type and transgenic plants.

C. The primary root of 8-d-old wild type and CEL over-expression transgenic plants on MS medium.

D. The primary root length of plants shown in (C). Presented are means ± SD (n = 3, >50 seeds per replicate experiment). There was no significant difference between wild type and transgenic plants.
